# Serum Estradiol and 20 Site-Specific Cancers in Women: Mendelian Randomization Study

**DOI:** 10.1210/clinem/dgab713

**Published:** 2021-10-03

**Authors:** Susanna C Larsson, Siddhartha Kar, John R B Perry, Paul Carter, Mathew Vithayathil, Amy M Mason, Douglas F Easton, Stephen Burgess

**Affiliations:** 1 Unit of Cardiovascular and Nutritional Epidemiology, Institute of Environmental Medicine, Karolinska Institutet, 17177 Stockholm, Sweden; 2 Unit of Medical Epidemiology, Department of Surgical Sciences, Uppsala University, 75185 Uppsala, Sweden; 3 MRC Integrative Epidemiology Unit, Bristol Medical School, University of Bristol, BS8 2BN Bristol, UK; 4 MRC Epidemiology Unit, Institute of Metabolic Science, University of Cambridge School of Clinical Medicine, Cambridge Biomedical Campus, CB2 0QQ Cambridge, UK; 5 Department of Public Health and Primary Care, University of Cambridge, CB1 8RN Cambridge, UK; 6 Department of Medicine, University of Cambridge, CB2 0QQ Cambridge, UK; 7 MRC Cancer Unit, University of Cambridge, CB2 0XZ Cambridge, UK; 8 British Heart Foundation Cardiovascular Epidemiology Unit, Department of Public Health and Primary Care, University of Cambridge, CB1 8RN Cambridge, UK; 9 National Institute for Health Research Cambridge Biomedical Research Centre, University of Cambridge and Cambridge University Hospitals, CB2 0QQ Cambridge, UK; 10 Centre for Cancer Genetic Epidemiology, Department of Public Health and Primary Care, University of Cambridge, CB1 8RN Cambridge, UK; 11 Centre for Cancer Genetic Epidemiology, Department of Oncology, University of Cambridge, CB1 8RN Cambridge, UK; 12 MRC Biostatistics Unit, University of Cambridge, CB2 0SR Cambridge, UK

**Keywords:** cancer, estrogens, estradiol, Mendelian randomization

## Abstract

**Context:**

The causal role of endogenous estradiol in cancers other than breast and endometrial cancer remains unclear.

**Objective:**

This Mendelian randomization study assessed the causal associations of endogenous 17β-estradiol (E2), the most potent estrogen, with cancer risk in women.

**Methods:**

As primary genetic instrument, we used a genetic variant in the *CYP19A1* gene that is strongly associated with serum E2 levels. Summary statistics genetic data for the association of the E2 variant with breast, endometrial, and ovarian cancer were obtained from large-scale consortia. We additionally estimated the associations of the E2 variant with any and 20 site-specific cancers in 198 825 women of European descent in UK Biobank. Odds ratios (OR) of cancer per 0.01 unit increase in log-transformed serum E2 levels in pmol/L were estimated using the Wald ratio.

**Results:**

Genetic predisposition to higher serum E2 levels was associated with increased risk of estrogen receptor (ER)-positive breast cancer (OR 1.02; 95% CI, 1.01-1.03; *P* = 2.5 × 10^−3^), endometrial cancer overall (OR 1.09; 95% CI, 1.06-1.11; *P* = 7.3 × 10^−13^), and endometrial cancer of the endometrioid histology subtype (OR 1.10; 95% CI, 1.07-1.13; *P* = 2.1 × 10^−11^). There were suggestive associations with breast cancer overall (OR 1.01; 95% CI, 1.00-1.02; *P* = 0.02), ovarian cancer of the endometrioid subtype (OR 1.05; 95% CI, 1.01-1.10; *P* = 0.02), and stomach cancer (OR 1.12; 95% CI, 1.00-1.26; *P* = 0.05), but no significant association with other cancers.

**Conclusion:**

This study supports a role of E2 in the development of ER-positive breast cancer and endometrioid endometrial cancer but found no strong association with other cancers in women.

Estrogens are a class of steroid hormones with a fundamental role in a wide range of physiological processes, such as menstrual cycle regulation, reproduction, preservation of bone density, and modulation of brain function (1). 17β-estradiol (E2) is the most potent estrogen and has pro-oncogenic effects through increased cell proliferation and decreased apoptosis, mediated primarily by activation of the estrogen receptor (ER) alpha (1). Factors associated with higher lifetime estrogen exposure, such as early menarche, late menopause, and menopausal hormone therapy, are linked to increased risk of cancers of the breast (particularly ER-positive tumors) (2-6), endometrium (7, 8), and ovaries (particularly the endometrioid subtype) (9-11), whereas oral contraceptive use is linked to lower risk of endometrial and ovarian cancer (12). Nevertheless, whether estrogens specifically are largely responsible for the observed associations is not known as reproductive years are also associated with number of ovulations, and hormone therapy may be associated with confounding factors. Furthermore, the increased risk of breast cancer among women taking menopausal hormone therapy is mainly confined to estrogen-progesterone preparations (13), whereas estrogen-only preparations have weak (4) or no (13) association with risk of breast cancer. Although there is ample data on the associations of indirect measures of estrogen exposure and hormone therapy with risk of breast, endometrial, and ovarian cancer, studies on the causal role of endogenous estrogen levels for other cancers are scarce.

Mendelian randomization (MR) is a technique to provide evidence on causal relationships by exploiting genetic variants having a robust association with the exposure as instruments to predict the effect of the exposure on disease risk (14, 15). The advantage of an MR study over conventional observational studies is that confounding is diminished because genetic variants are randomly allocated at conception and thus normally not associated with environmental factors and self-selected behaviors. In addition, reverse causation is avoided because genes cannot be altered by disease status. Here, a 2-sample MR approach was applied to assess the potential causal associations of endogenous E2 levels with any and 20 site-specific cancers in women.

## Methods

### Genetic Instruments

As the primary genetic instrument for serum E2, we used the single-nucleotide polymorphism (SNP) rs727479 in *CYP19A1*, which encodes aromatase, an enzyme that converts androgens to estrogens. Aromatase is expressed in the gonads, placenta, adipose tissue, brain, and other tissues. Rs727479 and an SNP in complete linkage disequilibrium with this genetic variant in the *CYP19A1* gene (rs7173595) have previously been shown to be strongly associated with serum E2 levels in genome-wide association studies (GWAS) of postmenopausal women (16) and men (17, 18). This SNP was also associated with serum E2 in 25 502 premenopausal European women (<50 years of age and not reporting a hysterectomy or that menopause has occurred) in UK Biobank. We constructed a secondary genetic instrument for serum E2 that consisted of SNPs previously identified to be associated with this hormone in 206 927 men of European ancestry in the UK Biobank (18) and which were also associated with serum E2 at *P* < 0.05 in 25 502 premenopausal European women in the same cohort. Five SNPs met the criteria for the secondary genetic instrument. Table 1 shows the characteristics of the SNPs used for the primary and secondary genetic instruments for serum E2.

**Table 1.** Single-nucleotide polymorphisms used as instrumental variables for serum E2 levels in the primary and secondary genetic instrument

						Association with E2 in men^a^			Association with E2 in premenopausal women^b^		
Instrument	SNP	Chr	Gene	EA	OA	Beta	SE	*P* value	Beta	SE	*P* value
Primary	rs727479	15	CYP19A1	A	C	1.390	0.120	8.2 × 10^-30^	0.014	0.006	0.011
Secondary	rs1260326	2	GCKR	C	T	0.006	0.001	9.6 × 10^-11^	0.012	0.006	0.036
Secondary	rs45446698	7	CYP3A7	T	G	0.016	0.002	7.9 × 10^-14^	0.032	0.014	0.020
Secondary	rs34019140	14	ADAM6	G	A	0.012	0.001	6.9 × 10^-42^	0.011	0.006	0.043
Secondary	rs7173595^c^	15	CYP19A1	T	C	0.016	0.001	3.6 × 10^-72^	0.014	0.006	0.012
Secondary	rs727428	17	SHBG	C	T	0.006	0.001	1.8 × 10^-11^	0.025	0.005	<0.001

Abbreviations: Chr, chromosome; E2, 17β-estradiol; EA, effect allele (ie, the allele associated with higher serum E2 levels); OA, other allele; SE, standard error; SNP, single-nucleotide polymorphism.

^a^Effects estimates (beta coefficients and standard errors) represent the change in serum E2 in pg/mL from the genome-wide association study by Eriksson et al (17) (primary instrument) and the change in log-transformed E2 in pmol/L from the genome-wide association study by Ruth et al (18) (secondary instrument) per additional effect allele.

^b^Effects estimates (beta coefficients and standard errors) represent the change in serum E2 in log-transformed pmol/L per additional effect allele in premenopausal women in UK Biobank.

^c^In complete linkage disequilibrium with rs727479 (*CYP19A1*).

### Data Sources for Cancer

We obtained summary statistics GWAS data for breast, endometrial, and ovarian cancer from the Breast Cancer Association Consortium (19), a meta-GWAS of endometrial cancer (including data from the Endometrial Cancer Association Consortium, the Epidemiology of Endometrial Cancer Consortium, and UK Biobank) (20), and the Ovarian Cancer Association Consortium (21), respectively. Data from these consortia were extracted from the MR-Base platform (22).

We additionally estimated the associations of the E2-associated SNPs with any and 20 site-specific cancers in 198 825 unrelated women (37 to 73 years of age at the baseline assessment) of European descents in the UK Biobank cohort using logistic regression with adjustment for age and 10 genetic principal components, as described previously (23). Information on cancer outcomes was obtained from the national cancer registry, hospital episode statistics and death certification data, electronic health records, and self-reported information verified by interview with a nurse (Table 2). All analyses were restricted to pre- and postmenopausal women of European ancestry to minimize bias from population stratification.

**Table 2.** Definitions of site-specific cancer outcomes in the UK Biobank cohort

Cancer	ICD-9 codes	ICD-10 codes	Self-report (field 20001)	Cancer histology
Breast & gynecological cancers				
Breast cancer	174, 175, V10.3	C50, Z85.3	1002	
Endometrial/uterine cancer	179, 182, V10.42,	C54, C55, Z85.42	1040	
Cervical cancer	180, V10.41	C53, Z85.41	1041	
Ovarian cancer	183.0, 183.2, 183.8, 183.9, V10.43	C56, C57.0, C57.4, Z85.43	1039	
Blood cancers				
Non-Hodgkin lymphoma	200, 202.0, 202.1, 202.2, 202.7, V10.71	C82, C83, C84, C85, C86, C88.0, C88.4, Z85.72	1053	
Leukemia	204, 205, 206, 207, 208, V10.6	C91, C92, C93, C94.0, C94.2, C94.3, C94.4, C94.8, C95, Z85.6	1048, 1055, 1056, 1074	
Multiple myeloma	203.0, 203.1	C90.0, C90.1	1050	9732, 9733
Digestive system cancers				
Colorectal cancer	153, 154.0, 154.1, V10.05, V10.06	C18, C19, C20, Z85.038, Z85.048	1020, 1022, 1023	
Pancreatic cancer	157	C25, Z85.07	1034	
Esophageal cancer	150, V10.03	C15, Z85.01	1017	
Stomach cancer	151, V10.04	C16, Z85.028	1018	
Biliary tract cancer	155.1, 156.0	C22.1, C23, C24	1025	
Liver cancer	155.0	C22.0	1024	8170, 8171, 8172, 8173, 8174, 8175
Urinary tract cancers				
Bladder cancer	188, 189.1, 189.2, V10.51, V10.53	C67, C65, C66, Z85.51, Z85.54, Z85.53	1035	
Kidney cancer	189.0, V10.52	C64, Z85.528	1034	
Other cancers				
Melanoma	172, V10.82	C43, Z85.820	1059	
Lung cancer	162, V10.1	C33, C34, C39.9, Z85.1	1001, 1027, 1028, 1080	
Head and neck cancer	140, 141, 142, 143, 144, 145, 146, 147, 148, 149, 160, 161, V10.01, V10.02, V10.21, V10.22	C00, C01, C02, C03, C04, C05, C06, C07, C08, C09, C10, C11, C12, C13, C14, C30, C31, C32, Z85.21, Z85.22, Z85.81	1006, 1007, 1009, 1004, 1010, 1011, 1012, 1077, 1078, 1079, 1005, 1015, 1016	
Brain cancer	191, 192.0, 192.1, 192.2, 192.3, V10.85	C70, C71, C72.0, C72.3, Z85.841	1031, 1032, 1033	
Thyroid cancer	193, V10.87	C73, Z85.850	1065	

The Self-report and Cancer histology columns provide the internal UK Biobank codes used to define each outcome (available at https://biobank.ctsu.ox.ac.uk/crystal/coding.cgi?id=3 and https://biobank.ctsu.ox.ac.uk/crystal/coding.cgi?id=38).

Abbreviations: ICD, international classification of diseases.

All studies have been approved by a relevant ethical review board, and participants have provided informed consent. The MR analyses were approved by the Swedish Ethical Review Authority.

### Statistical Analysis

The associations of serum E2 instrumented by rs727479 in the *CYP19A1* gene region with the cancer outcomes were estimated using the Wald ratio method. For the MR analyses of serum E2 instrumented by 5 SNPs, 3 MR methods with different assumptions were applied. These included the multiplicative random-effects inverse variance weighted, weighted median, and MR-Egger methods (24). Effect estimates (beta coefficients and standard errors) for the SNP-E2 associations were obtained from UK Biobank (Table 1). All reported odds ratios (OR) of cancer were scaled per 0.01 unit increase in log-transformed serum E2 levels in pmol/L. Results were deemed statistically significant at the Bonferroni-corrected threshold of *P* < 0.0025 (*P* = 0.05/20 site-specific cancers). Associations with a *P* value between 0.0025 and 0.05 were regarded suggestive. The MR-Base platform (22) and Stata (StataCorp, College Station, Texas) were used for the MR analyses based on data from consortia and UK Biobank, respectively.

### Pleiotropy Assessment

The MR-Base platform (22) and PhenoScanner database V2 (25) were utilized to assess pleiotropic associations of the E2-related SNPs with other phenotypes, including potential confounders and mediators (ie, other sex hormones, reproductive factors, body mass index, and smoking).

## Results

**Figure 1.**
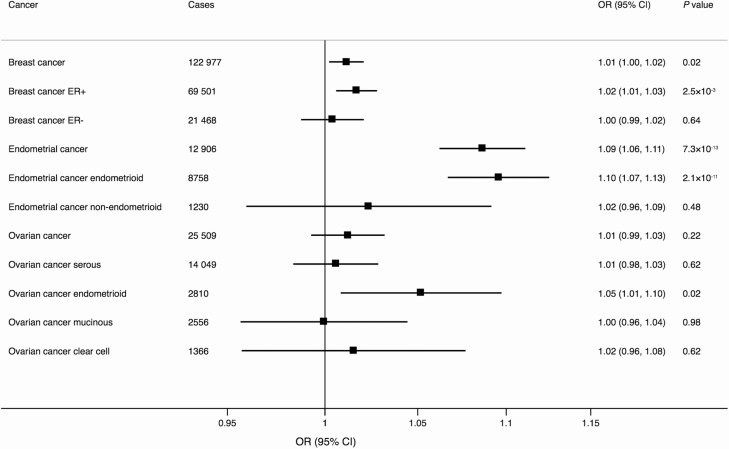
Associations of serum E2 levels instrumented by rs727479 in the *CYP19A1* gene region with breast, endometrial, and ovarian cancer and their subtypes based on data from consortia. The odds ratios are scaled per 0.01 unit increase in log-transformed serum E2 levels in pmol/L. The number of controls is 108 979 in the endometrial cancer meta-GWAS, 105 974 in the Breast Cancer Association Consortium, and 40 941 in the Ovarian Cancer Association Consortium. Abbreviations: E2, 17β-estradiol; ER+, estrogen receptor positive; ER-, estrogen receptor negative; OR, odds ratio.

**Figure 2.**
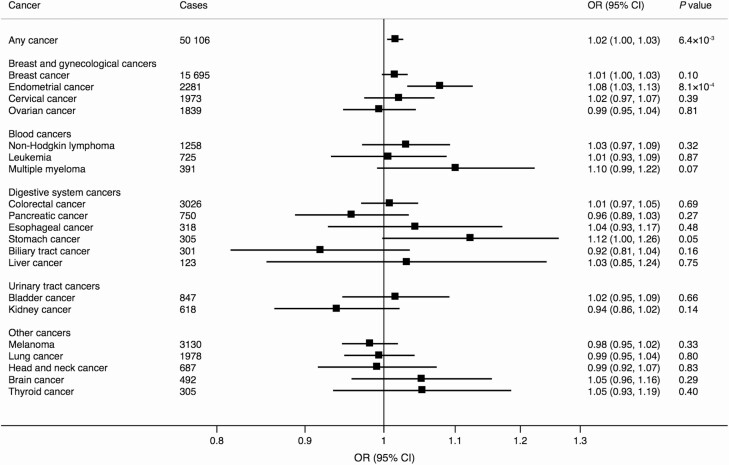
Associations of serum E2 levels instrumented by rs727479 in the *CYP19A1* gene region with any and 20 site-specific cancers in the UK Biobank cohort. The odds ratios are scaled per 0.01 unit increase in log-transformed serum E2 levels in pmol/L. Abbreviations: E2, 17β-estradiol; OR, odds ratio.

In the analyses based on data from the genetic consortia, genetic predisposition to higher serum E2 levels proxied by rs727479 in the *CYP19A1* gene was associated with increased risk of ER-positive breast cancer (OR 1.02; 95% CI, 1.01-1.03; *P* = 2.5 × 10^−3^) as well as with endometrial cancer overall (OR 1.09; 95% CI, 1.06-1.11; *P* = 7.3 × 10^−13^) and the endometrioid histology subtype (OR 1.10; 95% CI, 1.07-1.13; *P* = 2.1 × 10^−11^) (Fig. 1). There were suggestive associations with breast cancer overall (OR 1.01; 95% CI, 1.00-1.02; *P* = 0.02) and ovarian cancer of the endometrioid subtype (OR 1.05; 95% CI, 1.01-1.10; *P* = 0.02) (Fig. 1). In UK Biobank, genetic predisposition to higher serum E2 levels was associated with increased risk of any cancer and endometrial cancer, but the association with any cancer did not survive the Bonferroni-corrected significance level (Fig. 2). There was also a suggestive association with stomach cancer (OR 1.12; 95% CI, 1.00-1.26; *P* = 0.05) but no association with the other site-specific cancers (Fig. 2).

MR analyses of serum E2 instrumented by 5 SNPs showed no significant association with breast, endometrial, or ovarian cancer and their subtypes based on consortia data and 3 different MR methods (all *P* values > 0.05). Given the lack of association of this 5-SNP instrument with the positive control outcomes ER-positive breast cancer and endometrial cancer, we did not proceed with the corresponding analyses for 20 site-specific cancers using UK Biobank data, as these variants did not seem to be valid instruments for E2.

All SNPs but the *ADAM6* variant were associated with serum testosterone in men and women combined in UK Biobank. The *CYP19A1* and *GCKR* variants were further associated with fasting insulin, and the *GCKR* variant associated with body mass index. The *CYP3A7* variant was additionally associated with dehydroepiandrosterone sulfate, whereas the *SHBG* variant had further associations with dihydrotestosterone and body fat percentage.

## Discussion

This is the first MR investigation of the potential causal role of endogenous E2 levels for any cancer and a broad range of site-specific cancers. Our findings based on a genetic variant in the *CYP19A1* gene provide support that elevated serum E2 levels are causally linked to higher risk of ER-positive breast cancer and endometrial cancer, particularly of the endometrioid histology, suggesting a role of E2 in hormone-sensitive cancers. We found suggestive evidence that higher serum E2 levels may increase the risk of endometrioid ovarian cancer and stomach cancer. Serum E2 levels were not significantly associated with any other site-specific cancer but showed a suggestive positive association with risk of any cancer.

Our findings based on the *CYP19A1* variant corroborate the results of a pooled analysis of 4998 endometrial cancer cases and 8285 controls from 10 studies in the Epidemiology of Endometrial Cancer Consortium (26) as well as a study based on 6608 endometrial cancer cases and 37 925 controls from 4 studies (16). In those studies, each additional rs727479 A allele was associated with an 8% (26) and 15% (16) higher odds of endometrial cancer. Rs727479 and a correlated SNP (rs749292) in the *CYP19A1* gene region have also been reported to be associated with an increased risk of ovarian cancer in a small case-control study (367 cases and 602 controls) from Hawaii (27). Although serum E2 was not associated with ovarian cancer overall in the present analysis, our results suggested a positive association between serum E2 and the endometrioid subtype of ovarian cancer. *CYP19A1* gene polymorphisms, including rs727479 and rs3764221, have also been associated with an increased risk of lung cancer in small case-control studies (28, 29). We found no support for a positive association between serum E2 and lung cancer in our MR study. Data on E2-raising gene polymorphisms in relation to other cancers are scarce.

In the Women’s Health Initiative trial, there was suggestive evidence that estrogen plus progestin treatment might reduce the risk of colorectal cancer (6). The present MR study did not support an association between genetically predicted serum E2 and colorectal cancer risk in women. Whether estrogens or progesterone play a role in the prevention of colorectal cancer merits further study.

Our finding of a suggestive association between the *CYP19A1* gene variant and stomach cancer contrasts with observational studies which have shown that menopausal hormone therapy is associated with a lower risk of stomach cancer (30, 31). Furthermore, a nationwide cohort study of men with a diagnosis of prostate cancer found evidence of a reduced risk of stomach cancer in a male cohort exposed to estrogen (32). Given these inconsistent results and the weak evidence for causation in this investigation, it is possible that the suggestive association observed represents a chance finding.

The principal advantage of this study is the MR design, which reduced potential bias from confounders and reverse causality. Another important strength is that we evaluated the associations between serum E2 levels and a broad range of cancers of which most cancers have not previously been examined in relation to genetically predicted serum E2 levels. A limitation is that our analyses merely included women of European ancestry, thereby restricting the generalizability of our results to other populations. Another shortcoming is that the precision was low in the analyses of cancers with a limited number of cases (fewer than 1000 cases) and therefore we may have overlooked weak associations. Finally, higher genetically predicted E2 levels were associated with lower serum testosterone. Given that genetically predicted serum testosterone is positively associated with breast and endometrial cancer risk (18), the risk estimates for the associations between genetically predicted serum E2 and these cancers may be attenuated. Other serum E2-associated SNPs used in the secondary instrument were also associated with serum testosterone as well as with dehydroepiandrosterone sulfate, dihydrotestosterone, or body mass index. Thus, the lack of associations of serum E2 proxied by the secondary genetic instrument consisting of 5 genetic variants may be related to pleiotropy.

In conclusion, these MR findings support a causal role of endogenous E2 levels in ER-positive breast cancer and endometrioid endometrial cancer. Nevertheless, we found no evidence of a strong association of endogenous E2 levels with a broad range of other site-specific cancers in women.

## Data Availability

Some or all datasets generated during and/or analyzed during the current study are not publicly available but are available from the corresponding author on reasonable request.

## Abbreviations

E2: 17β-estradiol
ER: estrogen receptor
GWAS: genome-wide association studies
MR: Mendelian randomization
OR: odds ratio
SNP: single-nucleotide polymorphism

## Additional Information

***Disclosures:*** The authors have no conflict of interest to declare.
